# Detection of Zika virus in mouse mammary gland and breast milk

**DOI:** 10.1371/journal.pntd.0007080

**Published:** 2019-02-11

**Authors:** Jose Angel Regla-Nava, Karla M. Viramontes, Teodora Vozdolska, Anh-Thy Huynh, Tom Villani, Graeme Gardner, Michael Johnson, Pamela J. Ferro, Sujan Shresta, Kenneth Kim

**Affiliations:** 1 Division of Inflammation Biology, La Jolla Institute for Allergy & Immunology, La Jolla, California, United States of America; 2 Visikol, New Jersey, United States of America; 3 Texas Veterinary Medical Diagnostic Laboratory, College Station, TX, United States of America; 4 Department of Medicine, School of Medicine, University of California, San Diego, La Jolla, CA, United States of America; University of Texas Medical Branch, UNITED STATES

## Abstract

Clinical reports of Zika Virus (ZIKV) RNA detection in breast milk have been described, but evidence conflicts as to whether this RNA represents infectious virus. We infected post-parturient AG129 murine dams deficient in type I and II interferon receptors with ZIKV. ZIKV RNA was detected in pup stomach milk clots (SMC) as early as 1 day post maternal infection (dpi) and persisted as late as 7 dpi. In mammary tissues, ZIKV replication was demonstrated by immunohistochemistry in multiple cell types including cells morphologically consistent with myoepithelial cells. No mastitis was seen histopathologically. In the SMC and tissues of the nursing pups, no infectious virus was detected via focus forming assay. However, serial passages of fresh milk supernatant yielded infectious virus, and immunohistochemistry showed ZIKV replication protein associated with degraded cells in SMC. These results suggest that breast milk may contain infectious ZIKV. However, breast milk transmission (BMT) does not occur in this mouse strain that is highly sensitive to ZIKV infection. These results suggest a low risk for breast milk transmission of ZIKV, and provide a platform for investigating ZIKV entry into milk and mechanisms which may prevent or permit BMT.

## Author summary

Can Zika virus be transmitted from nursing mothers to their children via breast milk? Only 4 years have passed since the Zika virus outbreak in Brazil, and much remains to be understood about the transmission and health consequences of Zika infection. To date, some case reports have detected Zika virus RNA in the breast milk of infected mothers, but the presence of a virus’ RNA does not mean that intact virus is present. Milk also contains many natural defense components against infection, so even intact virus carried in breast milk may not be infectious to a child. Here we used a mouse that is genetically engineered to be highly susceptible to Zika infection, and tested whether 1) we could find intact virus in mouse breast milk and 2) infection was passed from mother to pups. We found very low levels of intact Zika virus in mouse breast milk, and found none of the nursing pups to be infected. The model of Zika virus breast milk infection developed in this study establishes a system by which we may learn whether Zika RNA in human breast milk is truly infectious to children, and how Zika virus may enter the milk.

## Introduction

Zika virus (ZIKV) is an enveloped virus with a positive-sense, single-stranded RNA genome [1]. For over half a century, this flavivirus was regarded as an arbovirus leading to self-limiting, febrile disease. However, confirmation of or association with new syndromes, including teratogenesis, adult Guillain Barre Syndrome, genital persistence, and sexual transmission, have begun to emerge since the 2015–2016 Brazil ZIKV outbreak. Due to devastating outcomes associated with infection of the developing brain and ZIKV’s apparent ability to cross intact mucosae [2–4], a key question arises: can ZIKV be transmitted by breast milk?

Reports of ZIKV RNA detection in breast milk are accumulating [5–10]. Although no epidemiologic data regarding ZIKV in lactating women are currently available, ZIKV RNA has been reported in breast milk from 3 [5, 9] to 33 [6] days after maternal onset of fever. Reports conflict as to whether isolated ZIKV RNA represents infectious virus [7]. In one study, cytopathic effect (CPE) could not be demonstrated in cells cultured with either of the breast milk samples from two mothers who nursed infected infants [9]. In two separate reports, CPE was seen upon culturing of cells with breast milk of mothers with uninfected nursing children [8, 10]. In another study, CPE was demonstrated in cells cultured with milk from a ZIKV-infected mother, and the nursing child was infected with an isolate with ZIKV genome identity of more than 99% between the infected mother and child [5].

Historically, the epidemiology and mechanisms of flavivirus breast milk transmission (BMT) have posed somewhat of a scientific enigma. Hepatitis C virus or Japanese encephalitis virus BMT has not been documented, whereas West Nile virus [11] and yellow fever vaccine strain [12] BMT have been reported. Dengue virus (DENV) infects approximately 390 million people annually and DENV RNA has been detected in breast milk [13], but reports of BMT are rare. Furthermore, in the 1970s, two studies also demonstrated that DENV and Japanese encephalitis virus were neutralized by the lipid fraction of breast milk [14, 15].

In this study, we explored a mouse model for BMT of ZIKV using AG129 mice that are deficient in both type I and II interferon (IFN) receptors, and represent a highly sensitive animal model of ZIKV challenge [16]. Following infection of AG129 dams with ZIKV on the date of parturition, viral RNA was detected in pup stomach milk clots (SMC) as early as 1 day post maternal infection (dpi) and as late as 7 dpi. In contrast, ZIKV NS2B immunofluorescent immunohistochemistry (IHC) and examination for CPE of inoculated Vero cells and focus forming assay did not demonstrate infectious virus in fresh milk or in nursing pups. Enzyme IHC provided evidence of intracellular viral replication *(i*.*e*. ZIKV NS2B expression) in cells morphologically consistent with epithelial cells, myoepithelial cells, and macrophages within the mammary gland. ZIKV NS2B expression was observed also in the SMC, and infectious particles were observed in fresh milk samples after 3 serial passages in Vero cells. The detection of potentially infectious ZIKV in the milk of this mouse model suggest that infectious virus may be present in human breast milk. However, BMT did not occur in this highly stringent ZIKV challenge system. These results suggest a low risk for human BMT of ZIKV, and set the stage for investigating ZIKV entry into milk and mechanisms by which BMT are prevented or permitted.

## Methods

### Mice and ethics statement

**Fig 1:**
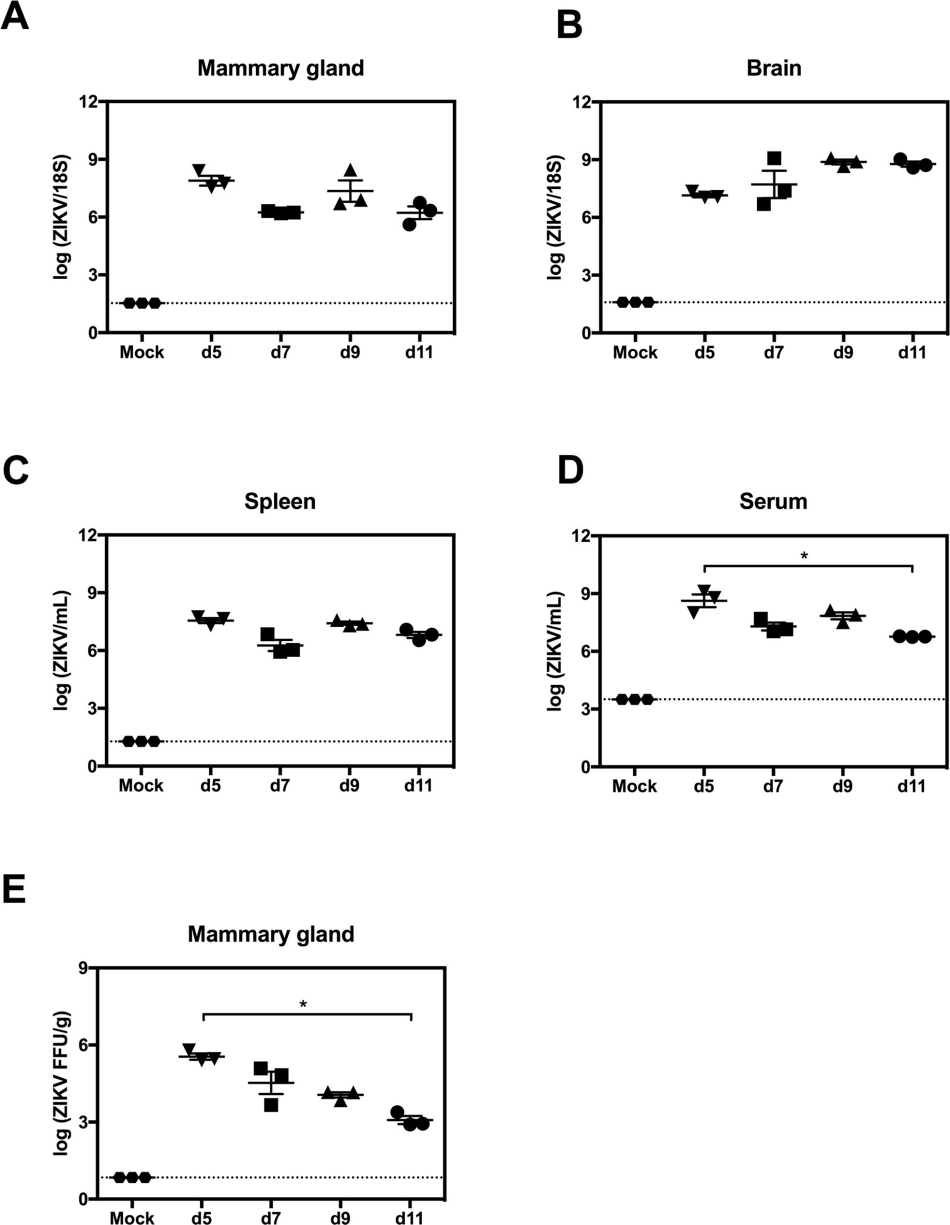
ZIKV replication in mammary glands of AG129 mice. Postpartum 8-week-old AG129 dams were retro-orbitally inoculated with 1 x 10^2^ FFU of ZIKV FSS13025 or 10% FBS-PBS as Mock within 24 hours of parturition. (A-D) Viral titers in mammary gland, brain, spleen and serum were determined via qRT-PCR at 5, 7, 9 and 11 days post infection (dpi). (E) Levels of infectious ZIKV in mammary gland were determined by FFA at 5, 7, 9 and 11 dpi. Negative controls were evaluated at 5 days after mock-infection (A-E). n = 3 mice per time point in each panel. Data represent two independent experiments.

**Fig 2:**
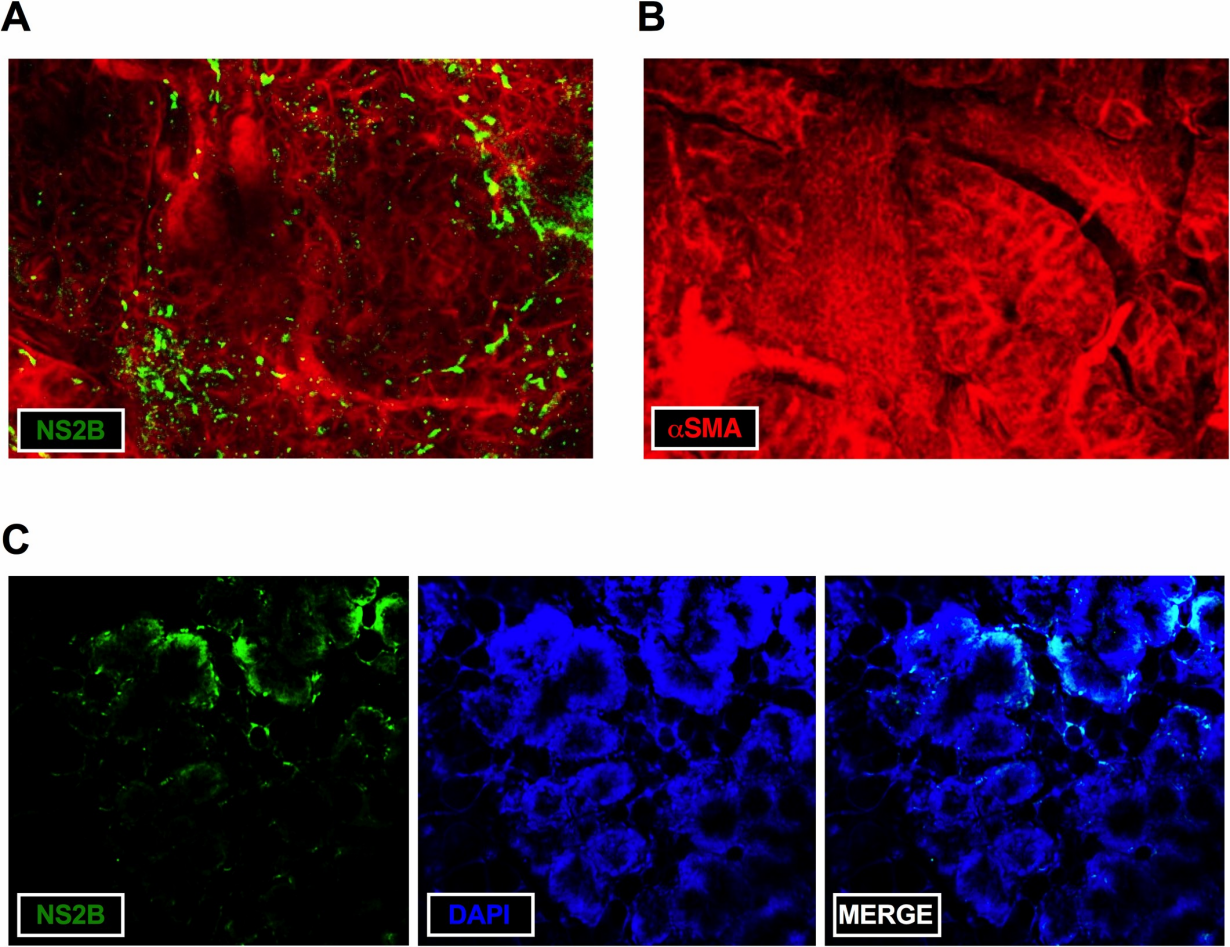
Max intensity z-projection of ZIKV-infected mammary tissue. Postpartum AG129 dams were inoculated via retro-orbital route with 1 x 10^2^ FFU of ZIKV FSS13025 or 10% FBS-PBS as Mock within 24 hours of parturition and sacrificed at 5 dpi. (A) Staining of mammary gland from ZIKV-infected mice with anti-αSMA (red) and anti-ZIKV NS2B antibodies (green). (B) Staining of mammary tissue from mock-infected mice with anti-αSMA (red) and anti-ZIKV NS2B antibodies (green). (C) Staining of mammary tissue from ZIKV-infected mice with anti-ZIKV NS2B (green) and DAPI (blue). Tissues were imaged in sequential mode at 2x digital zoom on a 10x air objective (20x overall). Representative data from 3 ZIKV-infected and 3 mock-infected mice are shown.

**Fig 3:**
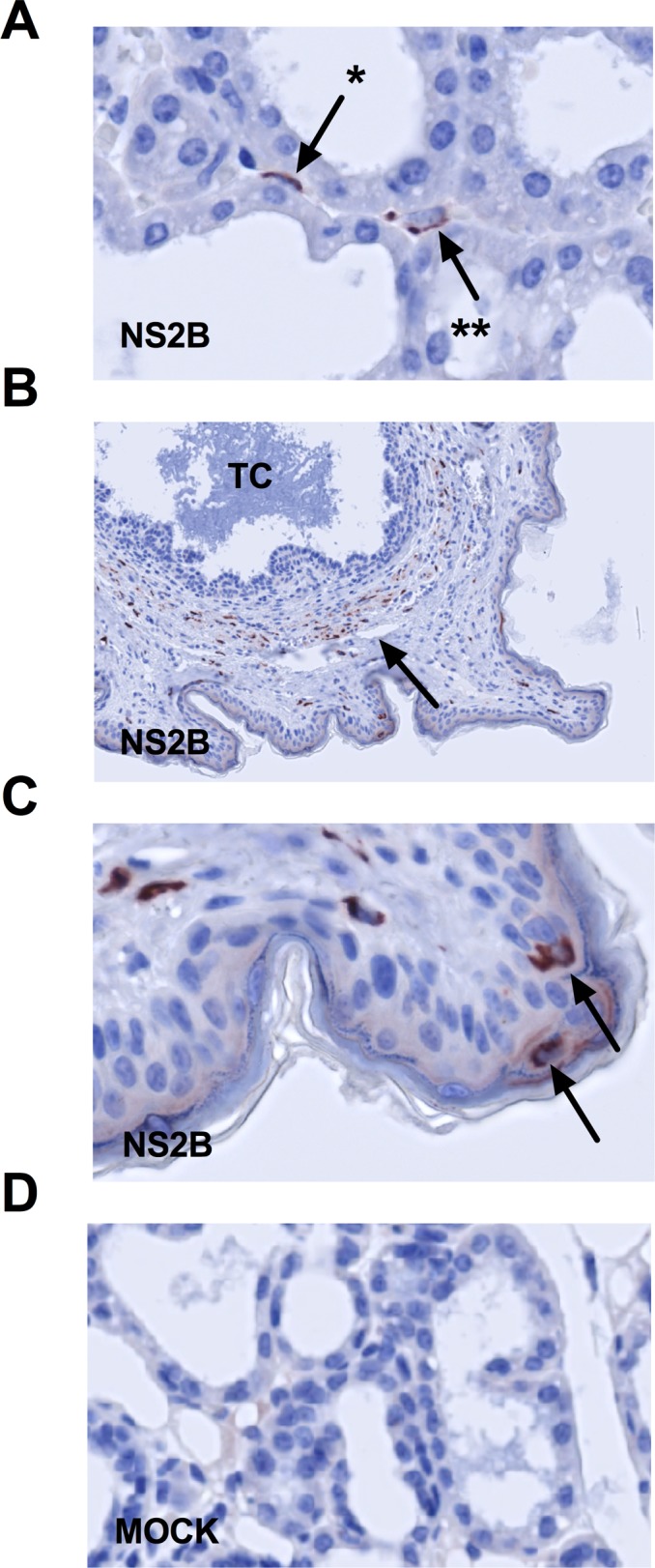
Immunohistochemical detection of ZIKV in mammary gland of AG129 mice. Postpartum AG129 dams were retro-orbitally inoculated with 1 x 10^2^ FFU of ZIKV FSS13025 or 10% FBS-PBS as Mock within 24 hours of parturition and sacrificed at 5 and 9 dpi. (A) Mammary gland was removed and ZIKV NS2B immunoreactivity was detected at 5 dpi. ZIKV NS2B immunoreactivity was detected in (*) myoepithelial cells and probable macrophages (**) in the interstitium. (B) At 9 dpi, many cells expressing ZIKV NS2B were seen in cells in the stroma surrounding teat canal. (C) Magnification of teat: There was strong ZIKV NS2B expression in some Langerhans cells. (D) Negative control mammary tissue from a mock-infected mouse is shown. Arrows indicate NS2B-expressing cells. TC, teat canal; n = 3 mice per group.

**Fig 4:**
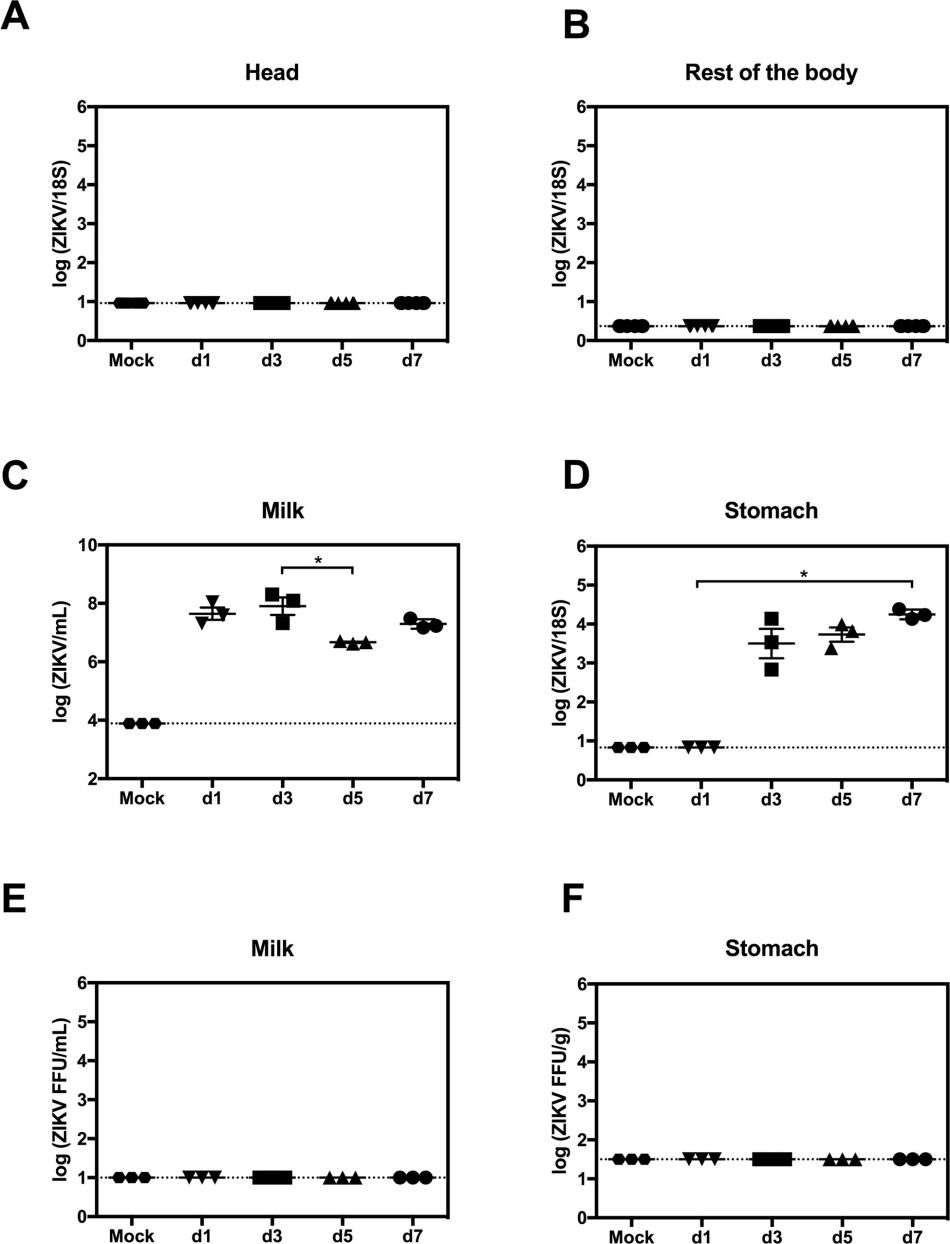
Transmission of ZIKV RNA in mouse breast milk. Postpartum AG129 dams were retro-orbitally inoculated with 1 x 10^2^ FFU of ZIKV FSS13025 or 10% FBS-PBS as Mock within 24 hours of parturition. Pups were sacrificed on d1, d3, d5 and d7 after birth. (A and B) Levels of ZIKV RNA in the head and the rest of the body were measured by qRT-PCR (n = 6 mice, each day). (C and D) ZIKV RNA levels in stomach milk clots (SMC) and stomach tissues were quantified via qRT-PCR (n = 3 mice, each day). (E and F) Presence of infectious ZIKV in SMC and stomach was assessed using FFA (n = 3 mice, each day). Data represent two independent experiments.

**Fig 5:**
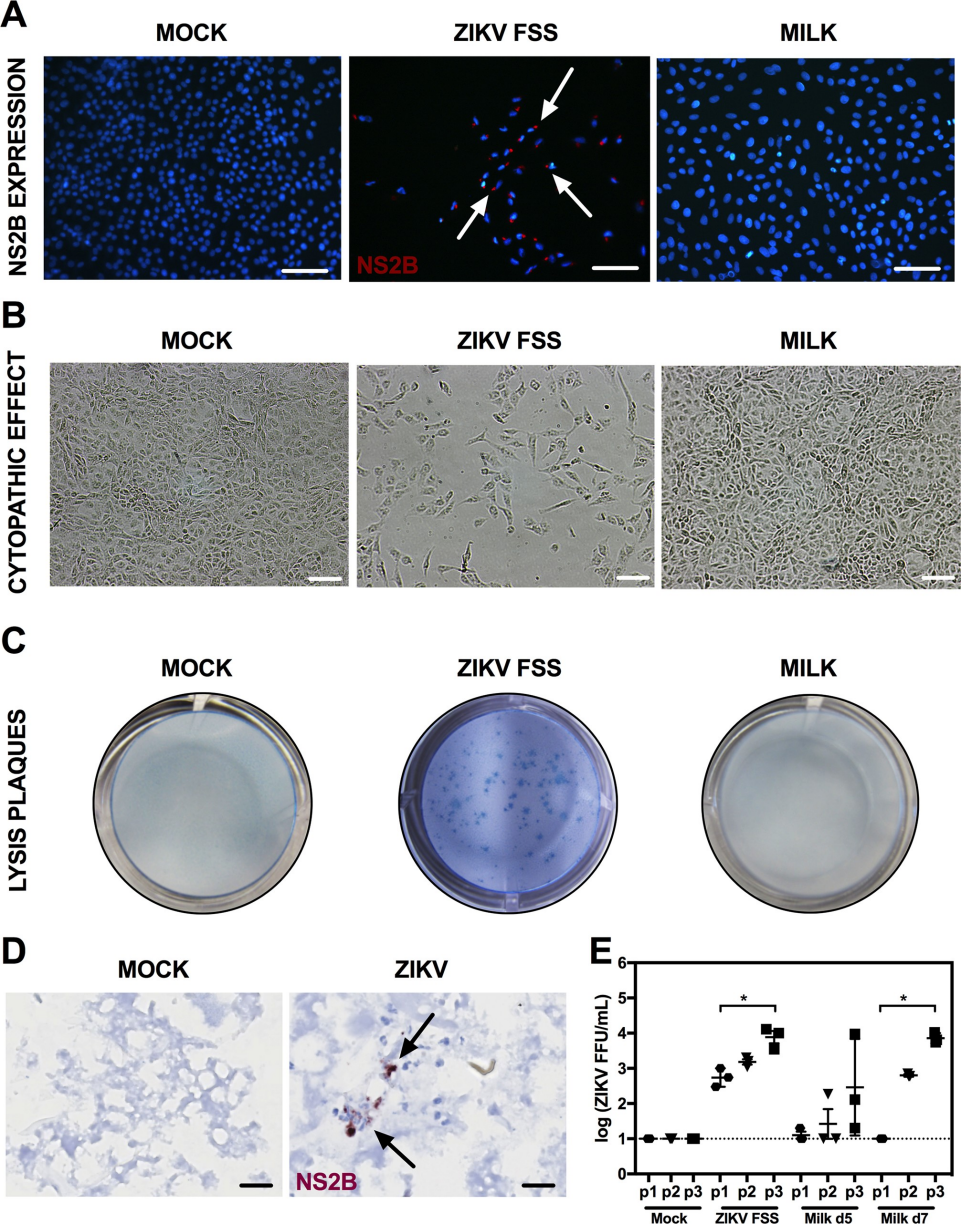
Infectivity of milk supernatant on vero cells. AG129 dams were retro-orbitally inoculated with 1 x 10^2^ FFU of ZIKV FSS13025 within 24 hours of parturition. (A-C) Vero cells were mock-infected as a negative control, infected with ZIKV FSS13025 at an MOI of 0.001 as a positive control, cultured with stomach milk clot (SMC) supernatant collected from the pup stomachs 3 days after birth, or (D) cultured with fresh milk obtained directly from dams at days 5 and 7 after infection; mock fresh milk was collected on day 5 after parturition. (A) Vero cells were fixed at day 5 after infection or culture with SMC supernatant. ZIKV NS2B protein (red) was labeled with a specific antibody. Nuclei were stained with DAPI (blue). Arrows indicate cells expressing ZIKV NS2B protein. (B) CPE and (C) plaques were visualized at at day 5 after infection or culture with SMC supernatant. Slides for immunofluorescence were examined on a Nikon/eclipse 80 microscope and CPE on an Eclipse TE300 microscopy and Nikon DXM1200C camera. (D) Postpartum AG129 mice were retro-orbitally inoculated with 1 x 10^2^ FFU of ZIKV FSS13025 or 10% FBS-PBS as mock within 24 hours of parturition. Pups were sacrificed on day 5 after birth. ZIKV NS2B expression was determined in SMC via IHC. Negative control SMC samples are obtained from pups born to mock-infected mice. Representative images from n = 10 mice per group are shown. (E) Levels of infectious ZIKV in the fresh milk samples were determined after serial passages in Vero cells using FFA. The Vero cell culture supernatants were collected at day 3 after culture with fresh milk, and this passaging was repeated for 2 additional rounds.; n = 3 mice per group.

129/Sv mice deficient in type I and type II IFN receptors (AG129) were bred and maintained at the La Jolla Institute for Allergy & Immunology (LJI) under standard pathogen free conditions. LJI has established an animal care and use program in compliance with The Public Health Service Policy on the Humane Care and Use of Laboratory Animals and maintains an animal welfare assurance with the Office of Laboratory Animal Welfare (OLAW). The animal care and use program is guided by the US Government Principles for the Utilization and Care of Vertebrate Animals Used in Testing, Research and Training and by the 8th edition of the Guide for the Care and Use of Laboratory Animals. As such, all research involving animals is reviewed and approved by the IACUC in accordance with The PHS policy on the Humane Care and Use of Animals and the 8th edition of The Guide. In addition, LJI’s animal care and use program is accredited by AAALAC International. All experiments involving these mice were approved by the Institutional Animal Care and Use Committee under protocol no. AP028-SS1-0615. Samples sizes: Fig 1 (1A to 1D: 3 pups per group from 3 separate mothers, 1E: 3 pups per group from 3 other separate mothers), Fig 2 (3 mothers per group), Fig 3 (3 mothers per group), Fig 4 (4A and 4B: 6 pups per group from 3 separate mothers, 4C and 4D: 3 pups per group from 3 separate mothers, 4E to 4F: other 3 pups per group from 3 separate mothers). Fig 5 (5A to 5C: images representative from 3 independent experiments, 5D: 3 mothers per group, 5E: 10 pups per group from 3 separate mothers). Animal experiments were not randomized or blinded.

### Viral strain, mouse infection, and cells

ZIKV strain FSS13025 (Cambodia, 2010) was obtained from the World Reference Center for Emerging Viruses and Arboviruses (WRCEVA). This strain was isolated from a pediatric case [17]. ZIKV was cultured using C6/36 *Aedes albopictus* mosquito cells as described previously [18]. Viral titers were determined by using baby hamster kidney (BHK)-21 cell-based focus forming assay (FFA) [19]. Eight-week-old female mice were infected retro-orbitally (r.o.) with 1 x 10^2^ focus forming units (FFU) of ZIKV FSS13025 in 200 μl 10% FBS/PBS. African green monkey kidney-derived Vero E6 cells were purchased from ATCC. Vero cells were grown in Dulbecco's modified Eagle's medium (DMEM, GIBCO) supplemented with 1% HEPES, 1% penicillin/streptomycin (GIBCO) and 10% fetal bovine serum (FBS, Gemini's BenchMark) at 37°C, 5% CO_2_.

### Milk samples

#### Stomach milk clots (SMC)

AG129 dams were either infected retro-orbitally with 1 x 10^2^ FFU of ZIKV FSS13025 within 24 hours of parturition or uninfected on the date of parturition. SMC was removed from the pup’s stomach.

#### Fresh milk

AG129 dams were either infected retro-orbitally with 1 x 10^2^ FFU of ZIKV FSS13025 within 24 hours of parturition or uninfected on the date of parturition. After 5 or 7 days post-infection, milking was performed. The pups were isolated from their mother for 6 hr in the same mouse box as the mother to allow milk to accumulate in the mammary gland. After separation, pups and dams were left together for 5 min. The dam was then removed and anesthetized with isoflurane. Milk was collected with a pipette in a microcentrifuge tube [20].

#### Isolation of the skim fraction from SMC and fresh milk

The SMC or fresh milk was separated into cream and skim milk by centrifugation (9,000 g for 10 minutes at 4 degrees), followed by removal of the cream [21]. Only the skim fraction was used in all analyses.

#### Serial passage of fresh milk in vero cells

The skim fraction of fresh milk was resuspended in 1 mL of DMEM and filtered with 0.22μM filtration unit (Millex, GV). These filtered preparations were inoculated into Vero cells for 3 days. After 3 days, the supernatants were passaged 3 additional times in Vero cells in order to increase the sensitivity of infection.

### qRT-PCR analysis of viral burdens

Mouse organs were collected in 800 μl RNA later (Ambion) and the tissues were transferred to 1% BMe/RLT buffer. Maternal mammary gland, brain, spleen, and the pup body minus the head and stomach were each placed in 800 μl. Before analysis, the skin of the head and rest of the body tissues were removed to avoid contamination from the mother’s saliva. SMC were removed from the pup’s stomach for separate analysis. The pups heads and stomachs tissues were processed in 250 μl (stomach was washed twice with PBS in order to remove remnant milk). The tissues were next homogenized for 3 minutes using Tissuelyser II (Qiagen Inc.) and centrifuged for 1 minute at 6,000 g. Tissue samples, SMC, and serum from ZIKV-infected mice were extracted with the RNeasy Mini Kit (tissues) or Viral RNA Mini Kit (serum and SMC) (Qiagen Inc.). Real-time qRT-PCR was performed using the qScript One-Step qRT-PCR Kit (Quanta BioSciences) and CFX96 TouchTM real-time PCR detection system (Bio-Rad CFX Manager 3.1). A published primer set was used to detect ZIKV RNA (Lanciotti, 2008). Fwd, 5’-TTGGTCATGATACTGCTGATTGC-3’; Rev, 5’-CCTTCCACAAAGTCCCTATTGC-3’ and Probe, 5’-6-FAM-CGGCATACAGCATCAGGTGCATAGGAG-Tamra-Q-3’. Cycling conditions were as follows: 45°C for 15 min, 95°C for 15 min, followed by 50 cycles of 95°C for 15 sec and 60°C for 15 sec and a final extension of 72°C for 30 min. Viral RNA concentration was determined based on an internal standard curve composed of five 100-fold serial dilutions of an *in vitro* transcribed RNA based on ZIKV FSS13025.

### IHC for ZIKV mammary tissue z-projection images and videos

The mammary gland was collected at 5 days post-infection (dpi) and was fixed in PFA for 24 hr at 4°C. ZIKV-infected tissues and mock-infected tissues were obtained. Tissues were processed and stained according to standard Visikol HISTO process (protocol.visikol.com) for antibody labeling. Tissues were immersed in Visikol Permeabilization Buffer at room temperature overnight. The following day, 2 mm thick tissue sections were processed through a series of washing steps of increasing methanol concentrations (50%, 80%, 100%), followed by permeation with 20% DMSO in methanol, and subsequently decreasing concentrations of methanol and back into PBS 1X. Tissues were then incubated in Visikol Penetration Buffer for 12 hr, washed with PBS, and incubated at 37°C in Visikol Blocking Buffer™ for 12 hr. Tissues were then transferred to microcentrifuge tubes for antibody labeling. The primary antibodies Smooth Muscle Actin (αSMA) (Invitrogen; goat polyclonal) and anti-ZIKV NS2B (GeneTex; rabbit polyclonal) were diluted at 1:100 in Visikol Antibody Buffer, and tissues were incubated at 37°C for 7 days. Tissue sections were washed in 1X Visikol Washing Buffer and then transferred to the secondary labeling solution. Secondary antibodies (DyLight 488 conjugated anti-goat and Alexa 594 conjugated anti-rabbit IgG-Invitrogen) were diluted at 1:200 in Antibody Buffer and the samples were incubated for another 3 days along with DAPI counterstain at 1:1000 dilution). Tissues were washed and cleared for imaging using (LSCM). For clearing, both control and infected tissues were dehydrated with sequentially increasing concentrations of methanol (i.e. 50% in PBS, 80% in H_2_O, 100%) for 30 min in each step, followed by incubation in Visikol HISTO-1 for 12 hours, and then into Visikol HISTO-2. Tissues were mounted in Visikol HISTO-2 and imaged using a Leica SP5 LSCM (laser scanning confocal microscope) using DAPI, Argon-488, and 594 nm lasers with 10X and 20X magnification objectives.

### Enzyme IHC, histopathology, and electron microscopy

The mammary gland was collected at 5, 7, 9 and 11 dpi; SMC were collected at 5 dpi; and mock-infected samples were prepared. Tissues were fixed in zinc formalin for 24 hr at room temperature. Tissues were processed for paraffin embedding, and sections for slides were cut at 4 μm thickness. For histopathologic evaluation, slides were stained with hematoxylin and eosin. For IHC, slides were microwaved in Antigen Unmasking Solution (Vector Laboratories), endogenous peroxidase activity was blocked by incubation in Bloxall (Vector Laboratories), and nonspecific protein binding was blocked by incubation in 10% goat serum. Slides were then incubated in rabbit anti-ZIKV NS2B antibody (Genetex) diluted at 1:100. ZIKV NS2B protein is a cofactor of the NS2B-NS3 protease which cleaves the viral polyprotein and is thus present during viral replication. Therefore, detection of NS2B serves as a marker of replicating virus as opposed to incomplete, phagocytosed, or degraded virions. Slides were then incubated sequentially in ImmPRESS HRP anti-rabbit IgG (Vector Laboratories), and NovaRED HRP Substrate (Vector Laboratories). IHC slides were also counterstained with hematoxylin. For each slide, the anti-ZIKV NS2B antibody staining was controlled with a slide using nonspecific Rabbit IgG (Vector Laboratories) substituted for the anti-ZIKV NS2B antibody, and control tissues from known infected and uninfected mice were included for each batch. A board-certified veterinary pathologist, who was blinded to each slide’s experimental conditions, read and scored each slide for immunoreactivity. Mammary gland slides were examined for mastitis by a pathologist. Bright field imaging was performed with a Zeiss Axio Scan.Z1 microscope and the images were acquired using Zen 2 software (Carl Zeiss). SMC were frozen on dry ice and sent to the Texas A&M Veterinary Medical Diagnostic Laboratory for transmission electron microscopy.

### Indirect immunofluorescence microscopy

To detect viral NS2B protein expression, Vero E6 cells were grown to 70% confluency on glass coverslips. Cells were either mock-infected or inoculated with ZIKV FSS13025 at a MOI of 0.001 or with SMC supernatant. The SMC was collected from the pup’s stomach on d3 after birth from AG129 dams that had been previously infected retro-orbitally with 1 x 10^2^ FFU of ZIKV FSS13025. The SMC was collected at day 3 post infection because this time point was the peak RNA viral burden in the SMC. At day 5 after SMC treatment, Vero cells were fixed in 4°C methanol and permeabilized with 0.1% Triton X-100. Protein blocking was performed with 10% goat serum, followed by incubation with anti-ZIKV NS2B antibody (Genetex) at 1:400 dilution. Coverslips were incubated with Alexa Fluor 594 (Invitrogen) at 1:300 dilution and then inverted onto glass slides for mounting. Imaging was performed by confocal microscopy.

### Statistical analysis

All data were analyzed with Prism software, version 7.0 (GraphPad Software) and expressed as means ± SEM. For viral burden and focus forming assay data, Krustal-Wallis test was used to compare more than two groups. This test was performed only in ZIKV-infected samples. Mock was not considered in the analysis. p<0.05 was considered a significant difference.

## Results

### Presence of infectious virus in the mammary glands of AG129 mice with ZIKV infection

To begin evaluating whether ZIKV could infect the mammary gland and be transmitted to breastfed infants, 8-week-old female AG129 mice were infected with ZIKV strain FSS13025. Viral burdens in several tissues were first assessed at 5, 7, 9 and 11 dpi via qRT-PCR. ZIKV RNA levels in the mammary glands were similar at the four time points (Fig 1A). As expected, high levels of ZIKV RNA were present in the brain, spleen, and serum with no significant difference among the four time points. With the exception of serum, there was a slight reduction on 5 dpi compared with 11 dpi (Fig 1B–1D), indicating ZIKV dissemination into tissues. To test for the presence of infectious virus in the mammary gland, we measured viral titers by FFA (Fig 1E). High levels of infectious ZIKV were present in the mammary gland at 5 dpi with a slight reduction in the subsequent days analyzed, demonstrating that ZIKV establishes productive infection in the AG129 mouse mammary glands.

### IHC for z-projection on ZIKV mammary tissue images and movies

To localize ZIKV replication within the mammary gland of AG129 mice, 8-week-old female AG129 mice were mock-infected or infected with ZIKV strain FSS13025, followed by visualization of ZIKV infection via laser scanning confocal microscopy. After clearing with Visikol HISTO, 800–1000 μm thick portions of the mammary gland were imaged under laser scanning confocal microscopy. Immunofluoresence staining was performed to assess expression of ZIKV NS2B, a marker for viral replication [22], and alpha smooth muscle actin (αSMA), present in myoepithelial cells. At 5 dpi, strong expression of NS2B and αSMA was detected in the mammary gland of ZIKV-infected AG129 mice (Fig 2A and 2B). The 3D images from this tissue (S1 and S2 Figs) and staining for ZIKV NS2B and DAPI (Fig 2C) showed similar results. Thus, ZIKV NS2B colocalizes with αSMA-expressing cells within the mammary glands of AG129 mice, suggesting myoepithelial cells as one of the cellular hosts of ZIKV in the mammary gland.

### Enzyme IHC detection of ZIKV replication in the mammary gland

To confirm ZIKV replication within the mammary gland of AG129 mice, tissues were fixed in Zinc formalin and then stained for expression of ZIKV NS2B at 5, 7, 9 and 11 dpi. No difference was observed among all times points, and we show 5 and 9 dpi as representative (Fig 3). NS2B expression was detected in cells morphologically consistent with mammary epithelial cells, myoepithelial cells, and interstitial macrophages (Fig 3A). NS2B expression in cells in the stroma surrounding the teat canal on a nipple cross section (Fig 3B) and teat Langerhans cells was also observed (Fig 3C). Additionally, histopathologic evaluation of these tissues revealed an absence of mastitis. Thus, ZIKV replicates locally in the mammary gland, and these enzyme IHC results in combination with z-projection images suggest that myoepithelial cells are major cellular hosts of ZIKV.

### ZIKV in mouse breastmilk does not induce systemic infection

Having established the presence of ZIKV RNA and infectious viral particles in the mammary gland, we proceeded to examine whether ZIKV was transmitted from infected mothers to neonates through breastfeeding. Neonatal heads, stomach tissues, SMC, and the rest of the bodies (without skin to avoid contamination from the mother’s saliva) were examined for the presence of ZIKV RNA by qRT-PCR. No ZIKV RNA was detected in the head and the rest of body in the neonates 1 to 7 days after birth (Fig 4A and 4B). However, viral RNA was present in SMC and stomach tissues at almost all time-points tested from 1 to 7 days after birth (Fig 4C and 4D). As ZIKV RNA does not necessarily indicate production of infectious virus, we next assessed for the presence of infectious ZIKV in SMC and stomach tissues via FFA. No infectious ZIKV particles were detected in SMC and stomach (Fig 4E and 4F). Thus, breastfeeding does not appear to be a significant route of ZIKV transmission into neonates in this mouse model.

### Infectivity of milk in Vero cells

To further assess the lack of infectious ZIKV in SMC, we inoculated SMC supernatant onto Vero cells. Infectivity of the SMC supernatant was assessed by immunofluorescence staining for ZIKV NS2B expression and CPE in the Vero cells, and plaque assay of the Vero culture supernatants. ZIKV NS2B expression and CPE were observed in the positive control cells infected with ZIKV. However, Vero cells inoculated with SMC supernatant did not show any NS2B protein expression or CPE (Fig 5A and 5B), and plaque assay confirmed the absence of infectious virus in the culture supernatant of SMC supernatant-treated Vero cells (Fig 5C). To assess whether ZIKV NS2B expression is observed in the breast milk were present in the SMC and might also infect the stomach tissue, 8-week-old female AG129 mice were infected with ZIKV strain FSS13025, followed by sacrificing of pups on d5 after postpartum and examination for the expression of ZIKV NS2B on the pup SMC and stomach tissue by IHC. Of 10 sampled pup stomachs, ZIKV NS2B expression surrounded nuclear material in SMC from 3 pup stomachs (Fig 5D). However, no ZIKV NS2B expression was detected in the full thickness of the gastric walls. These results suggest that replicating ZIKV may be passed in milk and is likely cell-associated; however, breast milk does not contain sufficient replication-competent ZIKV to initiate infection in cell culture and in IFN receptor-deficient mice.

Finally, we determined whether there is infectious virus present in fresh breast milk by FFA. To increase the sensitivity of infection in these samples, we performed serial passages in Vero cells of fresh breast milk collected at 5 and 7 days postpartum. Only one fresh milk sample collected at d5 showed a low infectivity in the first passage. However, we observed an increase of infectious particles at the second and third passage in both times points (Fig 5E).

## Discussion

In this study, we were able to detect ZIKV RNA in pup stomach milk clots and maternal mammary glands, and within the latter, ZIKV NS2B antigen localized to cells morphologically consistent with glandular epithelial cells, myoepithelial cells, and macrophages. ZIKV-permissive cells were also identified in the teat stroma and epidermis. Further, low levels of replicating virus were detected in fresh milk and ZIKV NS2B expression was detected in SMC samples. These results provide a framework for investigating ZIKV entry into the milk and raise the additional question of whether normal nursing-associated ingestion of maternal epidermal cells and blood may also play roles in ZIKV transmission.

We propose that infectious ZIKV may enter human breast milk but may be subsequently inactivated by endogenous or exogenous factors such as lipid, antimicrobial proteins, or gastric acid. Several studies have shown that the acidic pH and the digestive enzymes present in the stomach inactivate virus [23–25], and combine with mucus to form a chemical barrier to infection. Because dams were infected on the day of parturition, milk in this experiment should not have contained any ZIKV-neutralizing antibody. Recently in a rhesus macaques model, ZIKV RNA was present in saliva, another potential route of mucosal exposure [3], but no infectious virus was detected. Another study demonstrated that human breast milk inactivates ZIKV after prolonged storage [26]. Additionally, human breast milk has been reported to reduce the infectivity of HIV, HCV, and dengue virus. Thus, antiviral properties of breast milk may reduce BMT [21, 27].

Human viruses with known clinically relevant risk of BMT are cytomegalovirus (CMV) [28] and HIV-1 [27, 29–31]. Although mastitis is a risk factor for BMT of both viruses, most cases of BMT occur in the absence of mastitis. Further, most cases of CMV and HIV BMT involve a seroconverted mother rather than infection of a naïve mother in the nursing period. Infectious CMV has been isolated from up to 80% of infected breast milk samples, whereas infectious HIV has been extremely difficult to isolate from breast milk. DNA and RNA from other human viruses including herpesviruses, parvovirus, rubella virus, arboviral flaviviruses, and hepatitis viruses A, B and C have been detected in milk [32]. However, perhaps owing to low clinical relevance of BMT of these viruses, it is largely unknown whether detected nucleic acids were non-infectious viral genetic material or derived from neutralized virions.

After over two decades of research, the pathogenesis of HIV BMT remains poorly understood. It is estimated that BMT causes approximately 40% of mother-to-child transmission case of HIV. However, isolation of infectious virus from breast milk is rarely successful. HIV RNA, and rarely infectious virus, have been isolated from whey and cellular fractions of milk [33]. In contrast to CMV, viral loads in cellular fractions of milk correlate to transmission whereas loads in cell-free fractions do not. These findings have suggested that an intracellular location shields HIV from immune defenses such as lactoferrin, tenascin-C, defensins, and mucin [34]. Meanwhile, factors such as antibodies and HIV-gag-specific cytotoxic T lymphocytes may reduce cell-associated virus loads. Our early findings with ZIKV showed nursing mouse pups were not infected following ingestion of milk from infected dams. Therefore, the data are not sufficient to conclude that ZIKV infection can be passed via breastfeeding, and support early data suggesting the same for humans [8].

High ZIKV susceptibility of AG129 mice, which globally lack type I and type II IFN receptors, is often cited as a pitfall for many virology studies. However, in the current state of ZIKV science, in which it is unknown whether BMT is a clinical reality and there are no animal models of ZIKV entry into milk, a highly susceptible dam represents an excellent starting point to begin mechanistic manipulations which may reduce entry of viral RNA into milk. Furthermore, neonates, which are also deficient in IFN receptors, are a highly sensitive detection system for arranging conditions that may enable BMT. Indeed, the absence of infection in neonates in this study provides an early suggestion that infectious ZIKV is not easily transmitted through breast milk or other maternal-neonatal contact. It should also be noted that in humans, the tonsil is one of the first potential entry sites for orally ingested ZIKV [3], whereas mice do not have tonsils.

Because ZIKV is already known to have devastating consequences on the developing brain and there are both benefits to and substitutes for breastfeeding [35] it is imperative to fully understand the mechanisms which enable or prevent BMT. The results of this study provide a mouse model for investigating entry of ZIKV RNA into breast milk, and the pups provide a sensitive system for testing modulations which might permit BMT.

## Supporting information

S1 Fig AG129 dams were inoculated via retro-orbital route with 1 x 10^2^ FFU of ZIKV FSS13025 within 24 hours of parturition and sacrificed at 5 dpi. Staining of mammary gland from ZIKV-infected mice with anti-αSMA (red) and anti-ZIKV NS2B antibodies (green). Representative 3D images from 3 ZIKV-infected mice are shown.
(MOV)

S2 Fig AG129 dams were inoculated via retro-orbital route with 10% FBS-PBS as Mock within 24 hours of parturition and sacrificed at 5 dpi. Staining of mammary tissue from mock-infected mice with anti-αSMA (red) and anti-ZIKV NS2B antibodies (green). Representative 3D images from 3 mock-infected mice are shown.
(MOV)
